# *SLA2* is a prognostic marker in HNSCC and correlates with immune cell infiltration in the tumor microenvironment

**DOI:** 10.1007/s00405-023-08213-4

**Published:** 2023-09-09

**Authors:** Zhongbiao Wu, Chengkun You, Zhongyan Zhu, Weikun Wu, Jian Cao, Qiang Xie, Chengcheng Deng, Xinmei Huang, Shiping Hu

**Affiliations:** 1 Department of Otolaryngology, Jiangxi Hospital of Integrated Traditional Chinese and Western Medicine, 90 Bayi Avenue, Xihu District, Nanchang, 330003 Jiangxi China; 2 Department of Neurology, Pinghu Hospital of Traditional Chinese Medicine, Jiaxing, 314200 China; 3 Department of Rehabilitation, Jiangxi Hospital of Integrated Traditional Chinese and Western Medicine, Nanchang, 330003 China; 4 Department of Otolaryngology, Affiliated Hospital of Jiangxi University of Traditional Chinese Medicine, Nanchang, 330019 China; 5 Department of Otolaryngology, Jiangxi University of Traditional Chinese Medicine, Nanchang, 330004 Jiangxi China

**Keywords:** Head and neck squamous cell carcinoma (HNSCC), Src-like adaptor 2 gene (*SLA2*), Prognosis, Tumor microenvironment, Tumor immune cell infiltration

## Abstract

**Purpose:**

To investigate Src-like adaptor 2 gene (*SLA2*) expression in head and neck squamous cell carcinoma (HNSCC), its potential prognostic value, and its effect on immune cell infiltration.

**Methods:**

Through a variety of bioinformatics analyses, we extracted and analyzed data sets from the Cancer Genome Atlas (TCGA), Tumor Immune Estimation Resource (TIMER), and Gene Expression Profile Interaction Analysis (GEPIA) to analyze the correlation between *SLA2* and the prognosis, immune checkpoint, tumor microenvironment (TME) and immune cell infiltration of HNSCC, and to explore its potential oncogenic mechanism. To further explore the potential role of *SLA2* in HNSCC by Gene ontology (GO) functional annotation and Kyoto Encyclopedia of Genes and Genomes (KEGG) pathway analysis.

**Results:**

*SLA2* messenger ribonucleic acid (mRNA) levels were increased in HNSCC tumor tissues compared with normal tissues. In addition, we found that *SLA2* may be an independent prognostic factor for HNSCC, and high *SLA2* expression is associated with favorable prognosis in HNSCC. *SLA2* expression was positively correlated with B cells, cluster of differentiation 8-positive T cells (CD8 + T cells), cluster of differentiation 4-positive T cells (CD4 + T cells), macrophages, neutrophil and dendritic cells infiltration. *SLA2* has also been shown to co-express immune-related genes and immune checkpoints. Significant GO term analysis by Gene Set Enrichment Analysis (GSEA) indicated that genes correlated with *SLA2* were located mainly in the side of membrane, receptor complex, secretory granule membrane, endocytic vesicle, membrane region, and endosome membrane, where they were involved in leukocyte cell–cell adhesion, response to interferon-gamma, and regulation of immune effector process. These related genes also served as antigen binding, cytokine receptor activity, phosphatidylinositol 3-kinase activity, peptide receptor activity, Src homology domain 3 (SH3) domain binding, and cytokine receptor binding. KEGG pathway analysis demonstrated that these genes related to *SLA2* were mainly enriched in signal pathways, such as hematopoietic cell lineage, cell adhesion molecules (CAMs), natural killer cell mediated cytotoxicity, measles, and chemokine signaling pathway.

**Conclusions:**

*SLA2* is increased in HNSCC, and high *SLA2* expression is associated with favorable prognosis. *SLA2* may affect tumor development by regulating tumor infiltrating cells in TME. *SLA2* may be a potential target for immunotherapy.

## Introduction

Head and neck squamous cell carcinoma (HNSCC), which develops from the mucosal epithelium of the mouth, pharynx and larynx, is the most common malignant tumor of the head and neck. It is generally found in the late stage, with a high degree of malignancy and a poor prognosis. At present, although tumor resection combined with chemotherapy and radiotherapy has improved the prognosis of patients to a certain extent, advanced cancer is still prone to metastasis and deterioration. Therefore, it is necessary to explore more effective treatment methods in advanced HNSCC patients, such as targeted therapy. However, in the traditional research of targeted therapy for malignant tumors, the molecular pathways seem to be too complex for researchers to fully understand. So far, no effective biomarkers have been found [1].

Therefore, in this study, a variety of bioinformatics methods were used to explore the potential oncogenic mechanism of Src-like adaptor 2 gene (*SLA2*) in HNSCC, and the Cancer Genome Atlas (TCGA), Tumor Immune Estimation Resource (TIMER), Gene Expression Profile Interaction Analysis (GEPIA) and LinkedOmics data sets were extracted and analyzed. The correlation between *SLA2* and tumor immune cell infiltration, immune checkpoint, immune-related genes and immune checkpoint blockade (ICB) was detected, Gene ontology (GO) and Kyoto Encyclopedia of Genes and Genomes (KEGG), pathway enrichment analyses were applied to investigate the potential functions of *SLA2*. These results provide new insights into the function of *SLA2* and new targets for the diagnosis and prognosis of HNSCC.

*SLA2* is an adaptor protein that negatively regulates T-cell receptor (TCR) signaling. Inhibition of T cell antigen receptor induces activation of activated T cell nuclear factor. It may act by linking zeta chain of T cell receptor-associated protein kinase 70 (ZAP70) and other signaling proteins to Casitas B-lineage lymphoma proto-oncogene (CBL), resulting in CBL-dependent degradation of signaling proteins. It is involved in the development of various tumors, such as non-small cell lung cancer, breast cancer, and pancreatic duct adenocarcinoma [2–4]. However, whether *SLA2* affects the prognosis of HNSCC has not been investigated.

In recent years, immunotherapy has been applied to the treatment of HNSCC patients, changing the treatment of HNSCC [5]. It is well known that tumor-infiltrating immune cells (TIICs) affect the immune system, deal with abnormal biological behaviors in a complex way, and play an important role in response to immunotherapy [6]. In addition, genes related to immune components can also affect immune function [7]. Moreover, cytokine and immune checkpoint blockade therapy has become a therapeutic strategy for various types of cancer, but no study has investigated whether *SLA2* overexpression affects the tumor immune microenvironment of HNSCC.

## Methods

### Data source and analysis of differential expressions

Data acquisition, preprocessing, and ethics statement Pan-cancer ribonucleic acid sequencing (RNA-seq) data were obtained from UCSC XENA database (https:// xenabrowser.net/ datap ages/) [8]. Level 3 RNA RNA-seq expression data and clinical data for HNSCC (502 HNSCC tissues vs. 44 normal adjacent cancer tissues) were downloaded from TCGA portal (https:// portal. gdc. cancer. gov/) [9]. Before analysis, transcripts per million reads (TPM) RNA-seq data were log transformed (Log2(TPM + 1)). We use TCGA to analyze the *SLA2* expression difference between HNSCC and normal head and neck tissues. As TCGA are open publicly available databases, the data collection from the databases was compliant with all applicable laws, regulations, and policies for the protection of human subjects, and all written informed consents were obtained from all subjects involved.

### Exploring the association of survival with *SLA2* in HNSCC

We performed univariate and multivariate Cox regression analyses to determine the appropriate terms for building nomograms. A forest was applied to display the *p* value, hazard ratio (HR), and 95% confidence intervals (95% CI) for each variable using the “forestplot” R package through R software. A nomogram was created according to the results of the multivariate Cox proportional hazards analysis to predict the total recurrence rate in 1, 3, and 5 years. The “rms” R package was used to evaluate the risk of recurrence for individual patients by the points associated with each risk factor. We extracted survival information for each sample in TCGA. Indicators such as overall survival (OS) and disease-specific survival (DSS) were used to elucidate the relationship betweens *SLA2* and the prognosis of HNSCC patients. Kaplan–Meier (KM) and log-rank tests were used for the survival analysis of HNSCC (*p* < 0.05), and survival curves were analyzed by the “survminer” and “survivor” R packages.

### GEPIA analysis

GEPIA is a Newly developed interactive web server. Its web site is http://gepia.cancer-pku.cn/index.html [10]. This database provides online analysis based on data from TCGA and the Genotype-Tissue Expression (GTeX) projects. In our study, we used the given TCGA expression data set to assess the difference of *SLA2* between HNSCC and normal head and neck tissues. In addition, we used GEPIA to analyze the prognostic significance of *SLA2* mRNA expression in HNSCC and draw the survival curve with log rank *p* value.

### Immune infiltration analysis

Single-sample Gene Set Enrichment Analysis (ssGSEA) was used to assess the infiltration of individual immune cell populations, e.g., T helper 2 cells (Th2 cells), cluster of differentiation 8 T cells (CD8 T cells), natural killer CD56dim cells (NK CD56dim cells), and others. The analysis was performed using Gene Set Variation Analysis (GSVA) package [11, 12]. The TIMER (https://cistrome.shinyapps.io/timer/) database was applied to explore the correlations between HNSCC and TIICs (Li et al. 2017) [13]. GSVA package of R was used to analyze the correlations between *SLA2* expression levels and the infiltrations of T cells, B cells, regulatory T cell (TReg), CD8 T cells, dendritic cell (DC), activated dendritic cell (aDC), Cytotoxic cells, effective memory T cell (Tem), Eosinophils, and NK CD56dim cells.

### Immune-checkpoint analysis

Sialic acid binding Ig-like lectin 15 (SIGLEC15), T cell immunoreceptor with Ig and ITIM domains (TIGIT), cytotoxic T lymphocyte-associated antigen-4 (CTLA4), cluster of differentiation 274 (CD274), hepatitis A virus cellular receptor 2 (HAVCR2), lymphocyte-activation gene 3 (LAG3), programmed cell death 1 (PDCD1), and programmed cell death 1 ligand 2 (PDCD1LG2) were selected to be immune-checkpoint-relevant transcripts, and the expression data of these eight genes were extracted. R package “ggplot2” “pheatmap” and “immuneeconv” were used to assess the expression of the immune-checkpoints and co-expression of *SLA2* with these immune-checkpoints. Potential immune checkpoint blockade response was predicted with the TIDE algorithm (Jiang et al. 2018) [14].

### Spearman correlation analysis of IC50 score and *SLA2* expression

RNA-sequencing expression profiles and corresponding clinical information for HNSCC were downloaded from the TCGA data set (https://portal.gdc.com). Predicting the chemotherapeutic response for each sample based on the largest publicly available pharmacogenomics database [the Genomics of Drug Sensitivity in Cancer (GDSC), https://www.cancerrxgene.org/] [15–17]. The prediction process was implemented by R package “pRRophetic”. The samples' half-maximal inhibitory concentration (IC50) was estimated by ridge regression. All the parameters were set as the default values. Using the batch effect of the combat and tissue type of all tissue, the duplicate gene expression was summarized as the mean value using the batch effect of the combat and tissue type of all tissue. Predicting the relative IC50 of cisplatin and methotrexate in patients with HNSCC.

### LinkedOmics analysis

LinkedOmics is a public website on http://www.linkedomics.org/login.php, which contains 32 TCGA cancer types of multiple omics data, including federation generated from the Clinical Proteomics Tumor Analysis Consortium [18]. We applied this online tool to identify and analyze *SLA2* co-expression genes in head and neck cancer cohort from TCGA, which contains 502 samples. The data from Linkfinder are signed and sorted; after that, GO analyses, including molecular function (MF), biological process (BP), cellular component (CC), and KEGG analysis, are performed by the GSEA method.

### Statistical analysis

Gene expression data were normalized by log2-transformation. Statistical analyses were conducted using R software (version4.0.2). The analysis of *SLA2* expression in TIMER and TCGA databases showed *p* value. The survival curve generated by GEPIA analysis showed *p* value. The Cox proportional hazards model, KM analyses, and log-rank test were conducted for all survival analyses. Spearman’s test or Pearson’s test was applied to analyze the correlation between two variables; *p* value < 0.05 was considered significant. R software (version 4.0.2) was used for statistical analysis.

## Results

### *SLA2* expression in pan-cancer and HNSCC patients

**Fig. 1:**
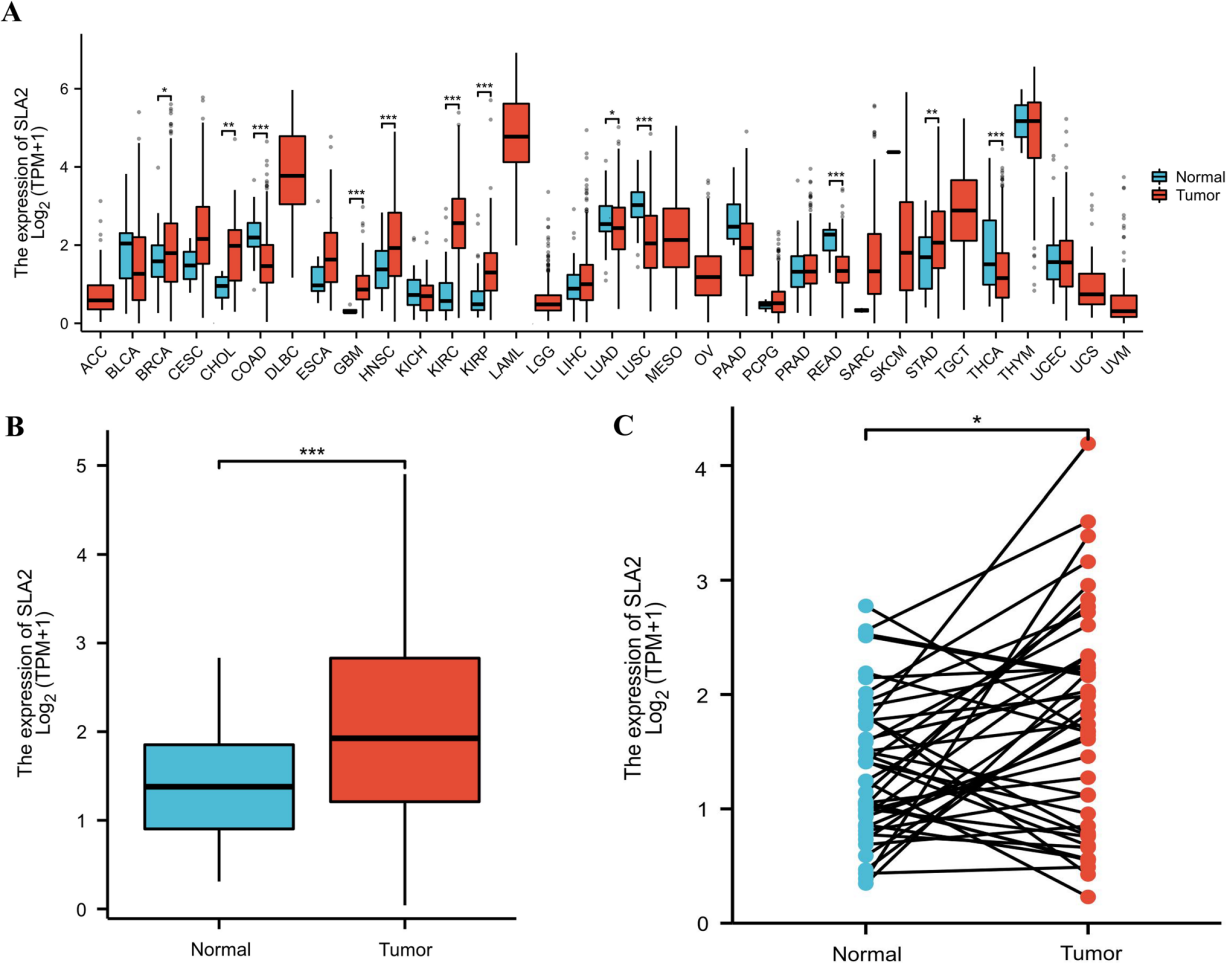
SLA2 expression in pan-cancer and HNSCC patients. **A** SLA2 expression levels in different tumor types. **B** SLA2 expression is significantly higher in HNSCC tissues than in normal tissues. ***p < 0.001. **C** SLA2 expression was also significantly higher in 43 HNSCC tissues than in matched normal adjacent tissue samples. *p < 0.05

Using the data from TCGA, we found that *SLA2* expression was significantly elevated in 19 of 33 cancer types, including kidney renal papillary cell carcinoma (KIRP), kidney renal clear cell carcinoma (KIRC), stomach adenocarcinoma (STAD), among others (Fig. 1A). Similarly, *SLA2* expression was significantly increased in HNSCC tissues compared with normal samples (Fig. 1B). It has the same result as in Fig. 1A, with a *p* value less than 0.001. For the 43 paired samples, *SLA2* expression was also significantly higher in cancer tissues than in matched normal adjacent tissue samples (Fig. 1C).

### The expression of *SLA2* Is associated with patients’ survival in HNSCC

**Fig. 2:**
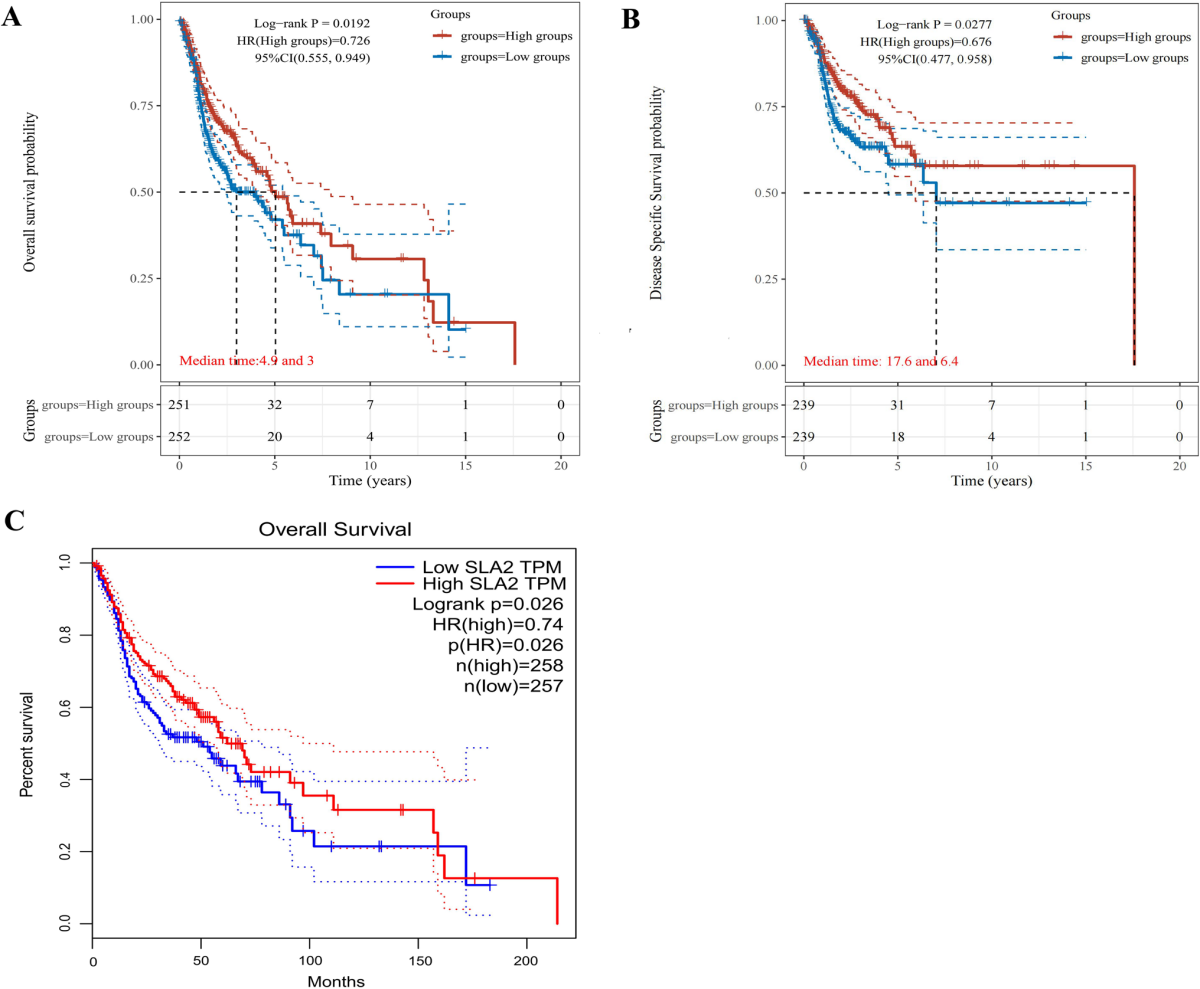
SLA2 expression level has potential diagnostic and prognostic values for patients with HNSCC. **A** KM survival curves for OS showed that patients with higher SLA2 mRNA expression had longer OS (*p* < 0.019). **B** KM survival curves for DSS showed that patients with higher SLA2 mRNA expression had longer DSS (*p* < 0.0277). **C** K–M survival curves for OS constructed from GEPIA

The survival data of *SLA2* expression in HNSCC patients were analyzed. KM survival curves were used to evaluate the relationship between the *SLA2* expression and survival outcomes. The cut-off value of the high and low *SLA2* expression group was set as the median. The results showed that patients with higher *SLA2* mRNA expression had longer OS (*p* < 0.019) (Fig. 2A), DSS (*p* < 0.0277) (Fig. 2B). Consequently, the expression of *SLA2* mRNA in HNSCC is associated with survival. The result from GEPIA is also consistent with our results (Fig. 2C).

### Prognostic potential of *SLA2* in HNSCC

**Fig. 3:**
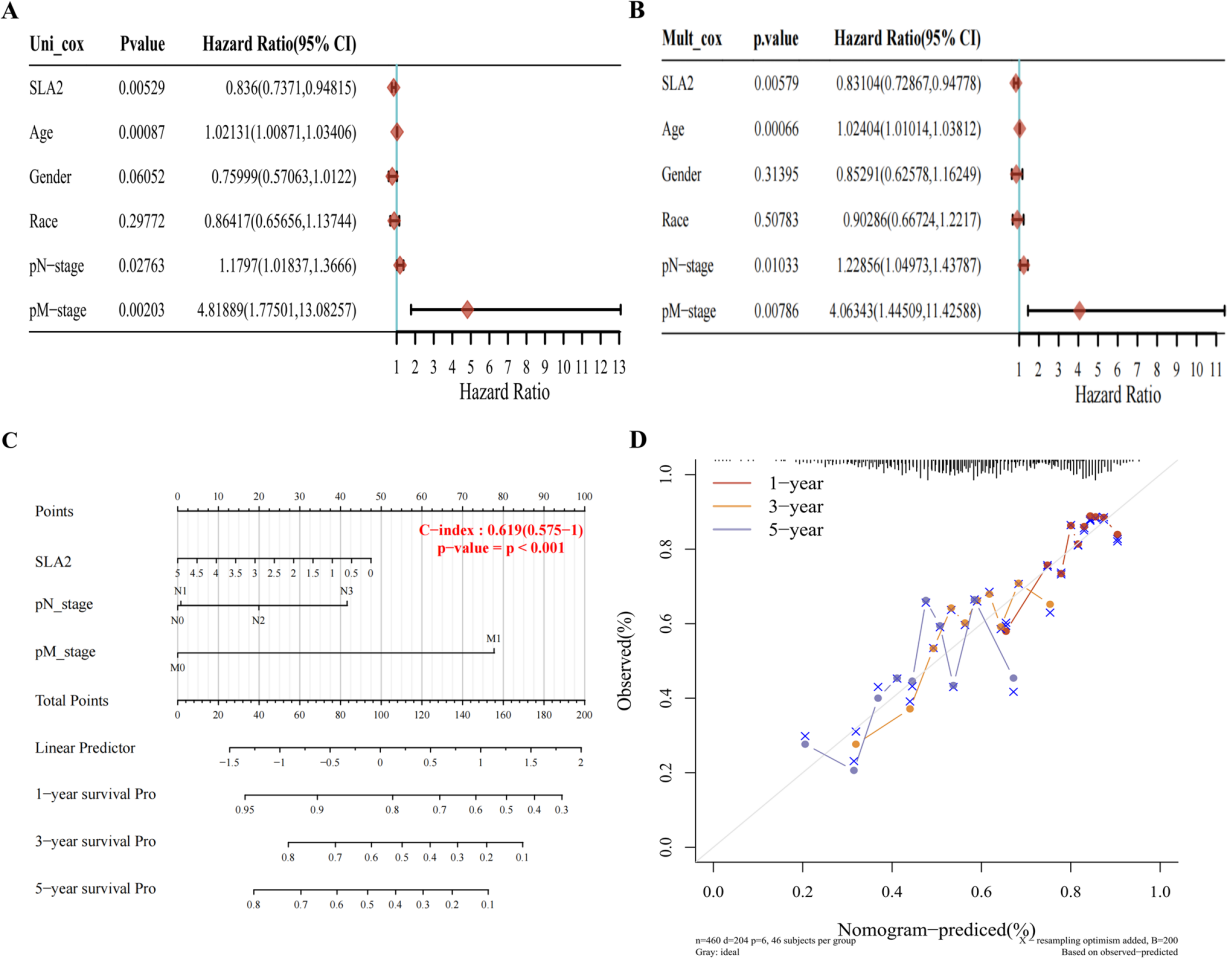
Prognostic efficacy of SLA2 expression levels in patients with HNSCC. **A** Univariate Cox regression analyses displayed using forest plots showed that SLA2 expression, pN-stage, and pM-stage were important factors to the prognosis of HNSCC. **B** Multivariate Cox regression analyses displayed using forest plots showed that SLA2 expression, pN-stage, and pM-stage were important independent factors to the prognosis of HNSCC. **C** Nomogram for predicting clinical outcomes associated with SLA2 expression in HNSCC patients. D Calibration curves showed that the nomogram performed well in estimating 1-, 3-, and 5-year OS

We then performed univariate and multivariate Cox regression analyses using several clinical features and *SLA2* expression level. Furthermore, univariate and multivariate Cox regression analyses illustrated that *SLA2* expression (*p* < 0.01), pN-stage (*p* < 0.05), and pM-stage (*p* < 0.01) were important independent factors to the prognosis of HNSCC (Fig. 3A, B). We further constructed a nomogram that combined only three independent prognostic factors (including *SLA2*, pN-stage, and pM-stage) to provide a quantitative guideline for clinicians to predict the probability of 1-, 3-, and 5-year OS in HNSCC patients (Fig. 3C). Each patient is given a total score through adding each prognostic parameter point, with a higher total score meaning a worse outcome for that patient. In addition, the calibration curves showed that the nomogram performed well in estimating 1-, 3-, and 5-year OS (Fig. 3D). Therefore, *SLA2* may be a potential diagnostic marker for HNSCC.

### Association between *SLA2* expression and immune cell infiltration and immune molecule expression levels

**Fig. 4:**
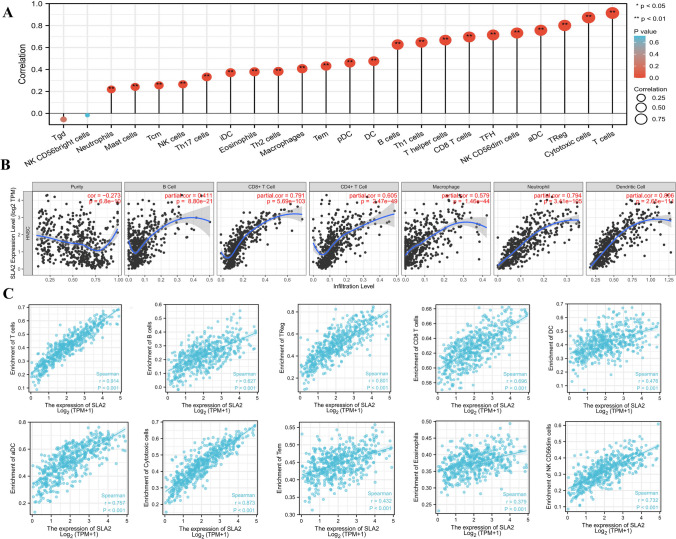
Relationship between SLA2 expression and immune cell infiltration in the tumor microenvironment. **A** SLA2 expression levels were positively correlated with numerous types of 24 immune cells. **B** Expression of SLA2 was positively correlated with the infiltration of (**B**), CD8 + , CD4 + , Macrophages, Neutrophils and dendritic cells determined using TIMER. C SLA2 expression levels were significantly positively correlated with T cells, B cells, TReg, CD8 T cells, DC, aDC, Cytotoxic cells, Tem, Eosinophils, and NK CD56dim cells enrichment levels (*p* < 0.01)

To understand the effects of *SLA2* expression on tumor microenvironment, immune infiltration analysis was performed using ssGSEA method. The correlations between enrichments of immune cell in Head and neck tissues and *SLA2* expression levels were calculated using Spearman correlation analyses. As shown in Fig. 4A, *SLA2* expression levels were positively correlated with numerous types of immune cells, e.g., T cells, Cytotoxic cells, TReg, aDC, NK CD56dim cells, follicular helper T cell (TFH), CD8 T cells, T helper cells, T helper 1 cells (Th1 cells), B cells, DC, plasmacytoid dendritic cells (pDC), Tem cells, Macrophages, Th2 cells, Eosinophils, interdigitating dendritic cell (iDC), Th17 cells, NK cells, central memory T cells (Tcm), Mast cells, and Neutrophils, all of which play an important role in anti-tumor immunity. Next, we further used the CIBERSORT algorithm to investigate the tumor-infiltrating immune cells, we found that the expression of *SLA2* was positively correlated with the infiltration of B cells, cluster of differentiation 8-positive T cells (CD8 + T cells), cluster of differentiation 4-positive T cells (CD4 + T cells), Macrophages cells, Neutrophils cells, and Dendritic cells (Fig. 4B). Similarly, the infiltrations of other immune cells in Head and neck tissues are observed, GSVA package of R was used to analyze the correlations between *SLA2* expression levels and the infiltrations of T cells, B cells, TReg, CD8 T cells, DC, aDC, Cytotoxic cells, Tem, Eosinophils, and NK CD56dim cells. As shown in Fig. 4C, *SLA2* expression levels were significantly positively correlated with the infiltrations of those immune cells (*p* < 0.01).

**Fig. 5:**
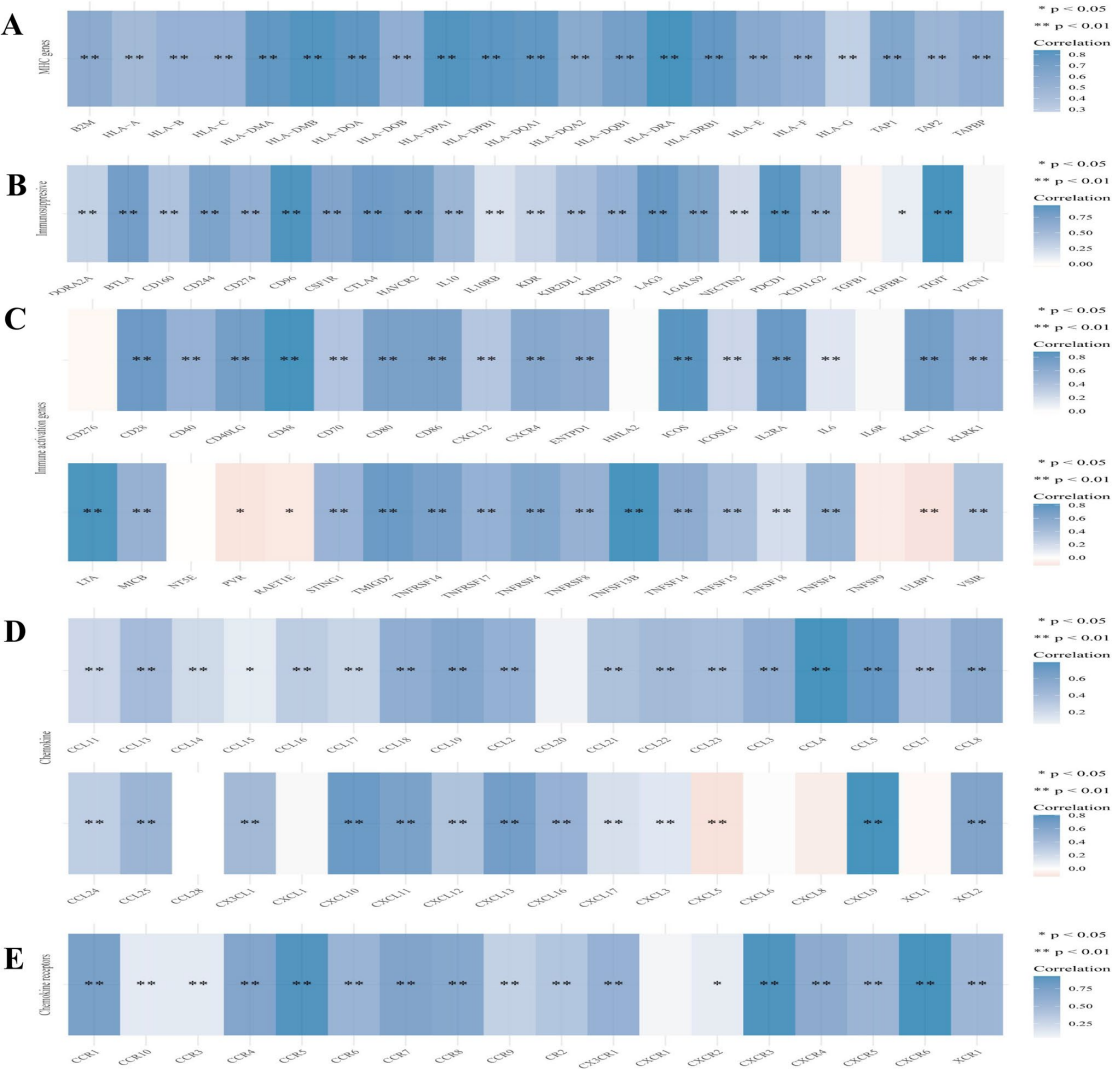
Co-expression of SLA2 with immune-related genes in HNSCC. **A** SLA2 positively co-expressed with all listed MHC genes. **B** SLA2 positively co-expressed with almost all immunosuppressive genes. **C** SLA2 had a positive relationship with most of the immune activation genes. **D** SLA2 had a positive correlation with some chemokine. **E** SLA2 had a positive correlation with some chemokine receptors

**Fig. 6:**
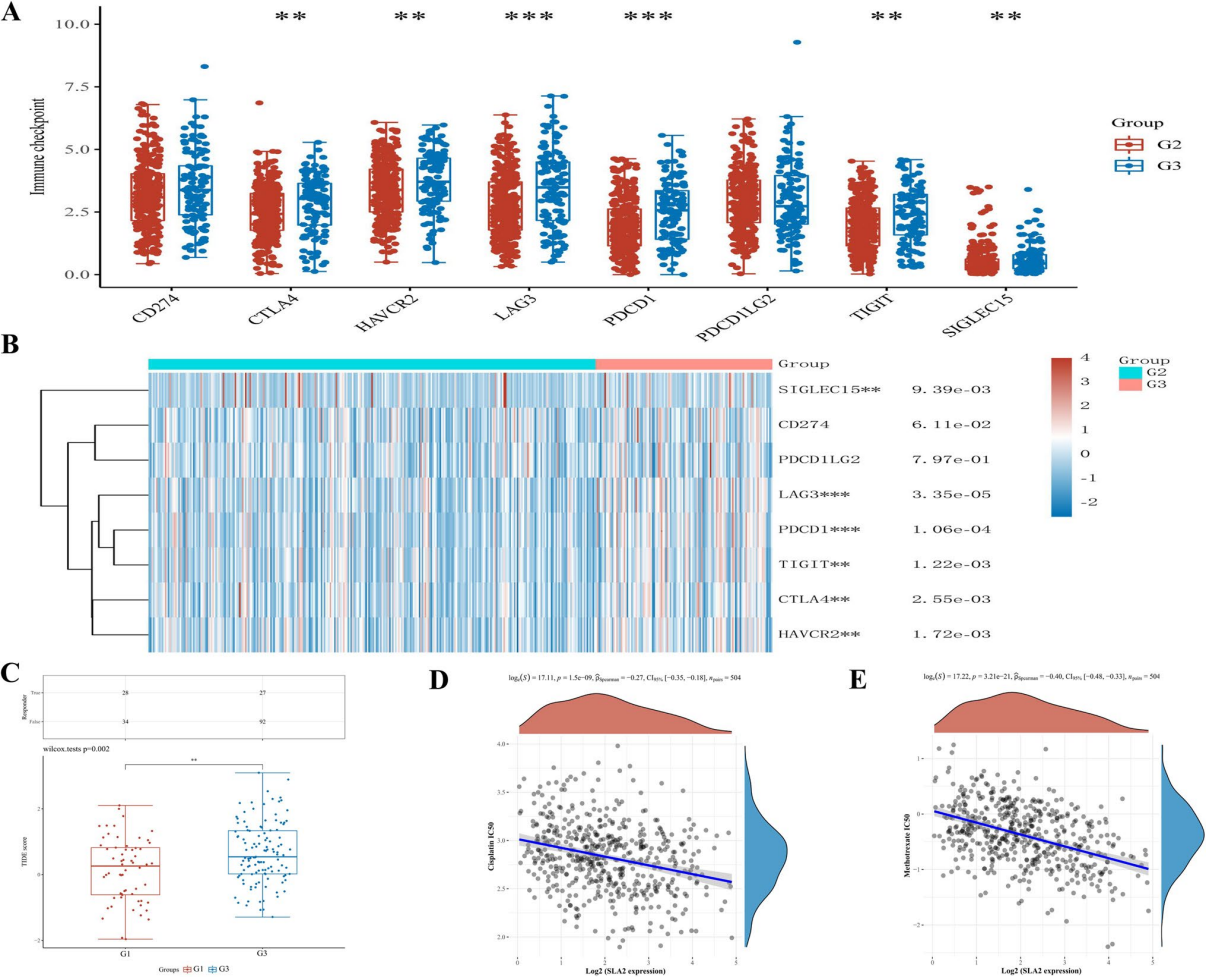
Expression of immune-checkpoints in HNSCC. **A**, **B** Expression of immune-checkpoints were upregulated in WHO grade III compared with WHO grade II of HNSCC. **C** HNSCC patients in WHO grade III had a better response to immune checkpoint blockade compared with HNSCC patients in WHO grade I. **D**, **E** IC50 of cisplatin and methotrexate was negatively correlated with SLA2 expression

We further performed gene co-expression analysis to evaluate the mechanisms that *SLA2* was correlated with the infiltration of immune cells in HNSCC. Major histocompatibility complex gene (MHC genes), immunosuppressive, immune activation genes, chemokine and chemokine receptor-related genes were assessed. The results showed that *SLA2* positively co-expressed with all listed MHC genes (Fig. 5A). Notably, almost all immunosuppressive genes positively co-expressed with *SLA2* (Fig. 5B). *SLA2* had a positive relationship with most of the immune activation genes, such as genes that encoding cluster of differentiation 48 T cells (CD48), cluster of differentiation 28 T cells (CD28), Interleukin-6 (IL6), C–X–C motif chemokine receptor 4 (CXCR4) (Fig. 5C). Similarly, *SLA2* had a positive correlation with some chemokine and chemokine receptors (Fig. 5D, E). The expression of immune-checkpoints, including CD274, CTLA4, HAVCR2, LAG3, PDCD1, PDCD1LG2, TIGIT, and SIGLEC15 (Fig. 6A), were further investigated in the World Health Organization (WHO) grade II and III of HNSCC. The results illustrated that LAG3 (*p* < 3.35e−05), PDCD1 (*p* < 1.06e−04), CTLA4 (*p* < 2.55e−03), HAVCR2 (*p* < 1.72e−03), TIGIT (*p* < 1.22e−03), and SIGLEC15 (*p* < 9.39e−02) were upregulated in WHO grade III compared with WHO grade II of HNSCC (Fig. 6B). In addition, we revealed that HNSCC patients in WHO grade III had a better response to immune checkpoint blockade compared with HNSCC patients in WHO grade I (Fig. 6C). Moreover, the results demonstrated that *SLA2* positively co-expressed with these immune-checkpoints (Table 1), indicating that *SLA2* may be a potential immunotherapy target.

**Table 1:** SLA2 co-expressed with immune-checkpoints

Genes	Cor	*P* value
CD274	0.607	4.54E-52
CTLA4	0.834	9.47E-132
HAVCR2	0.812	8.45E-120
LAG3	0.864	1.83E-151
PDCD1	0.92	9.01E-206
PDCD1LG2	0.557	1.80E-42
TIGIT	0.941	2.71E-238
SIGLEC15	0.338	6.00E-15

### Spearman correlation analysis of IC50 score and *SLA2* expression

RNA-sequencing expression profiles and corresponding clinical information for HNSCC were downloaded from the TCGA data set (https://portal.gdc.com). The results showed that IC50 of cisplatin and methotrexate was negatively correlated with *SLA2* expression (Fig. 6D, E).

### Enrichment analysis of *SLA2* neighborhood genes in HNSCC

**Fig. 7:**
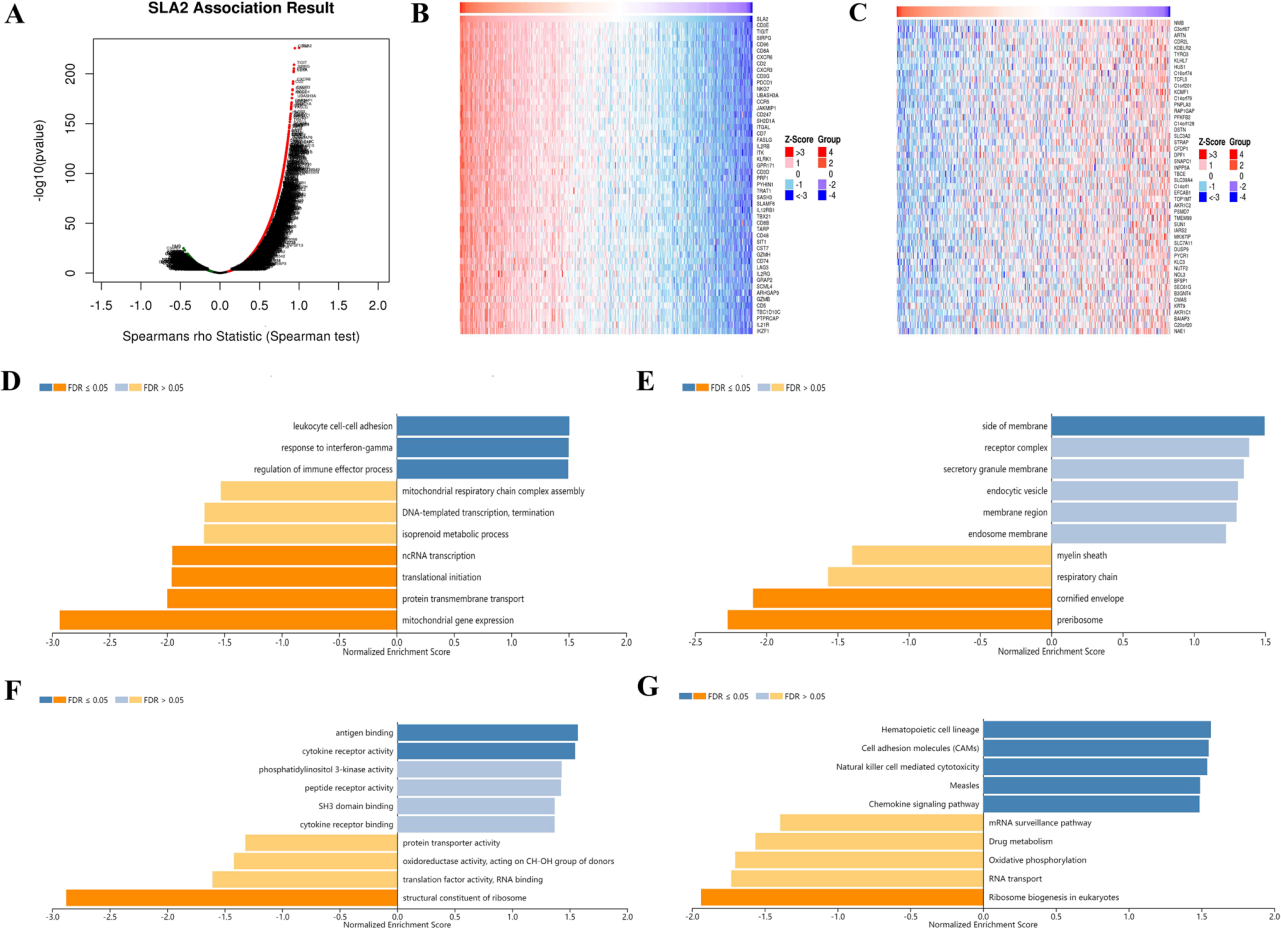
Genes differentially expressed in association with SLA2 in HNSCC, and enrichment analysis of the genes altered in the SLA2 neighborhood in HNSCC. **A** Volcano plot. **B** Heat maps of the top 50 genes positively correlated with SLA2. **C** Heat maps of the top 50 genes negatively correlated with SLA2. **D** Biological processes. **E** Cellular components. **F** Molecular functions. G KEGG pathway analysis

To study the functional network of *SLA2* neighborhood genes in HNSCC, we first identified *SLA2* neighborhood genes with LinkedOmics. The results are shown in a volcanic plot (Fig. 7A). In addition, the top 50 positively and negatively related genes were shown in the heat map, respectively (Fig. 7B, C). Significant GO term analysis by GSEA indicated that genes correlated with *SLA2* were located mainly in the side of membrane, receptor complex, secretory granule membrane, endocytic vesicle, membrane region, and endosome membrane, where they were involved in leukocyte cell–cell adhesion, response to interferon-gamma, and regulation of immune effector process. These related genes also served as antigen binding, cytokine receptor activity, phosphatidylinositol 3-kinase activity, peptide receptor activity, Src homology domain 3 (SH3) domain binding, and cytokine receptor binding (Fig. 7D–F). KEGG pathway analysis demonstrated that these genes related to *SLA2* were mainly enriched in signal pathways, such as hematopoietic cell lineage, cell adhesion molecules (CAMs), natural killer cell mediated cytotoxicity, measles, and chemokine signaling pathway (Fig. 7G).

## Discussion

HNSCC is the sixth most prevalent cancer in the world. It originates from different parts of the head and neck region, including the oral cavity, nasal cavity, nasopharynx, larynx, hypopharynx, and oropharynx. Squamous cell carcinoma (SCC) accounts for 90% of head and neck malignancies [19]. Due to the characteristics of delayed early diagnosis and high recurrence rate of HNSCC, about 50% of all patients will eventually have local or regional recurrence. Despite the progress in treatment, the 5-year survival rate is still very low, and there are still some patients with poor treatment effect and poor prognosis [20]. Therefore, it is of great significance to explore new genes related to the occurrence and development of HNSCC, and to find markers with higher specificity and sensitivity, especially other molecular mechanism targets, such as immune infiltration, for improving the treatment of HNSCC. The aim of this study was to investigate the diagnostic value of *SLA2* gene in HNSCC and its effect on tumor immune invasion. *SLA2* is an adaptor protein whose main function is to inhibit the activation of activated T cell nuclear factor induced by T cell antigen receptor [21]. Relevant studies have reported that *SLA2* expression mediates cell proliferation, colony formation and tumor formation by destroying receptors [3]. In conclusion, we can infer that *SLA2* may be involved in tumor formation and development by further affecting cell proliferation through the cellular microenvironment.

In our study, we found that *SLA2* was differentially expressed in various types of cancer and its corresponding normal tissues. A study indicates that *SLA2* may be involved in immune response for promoting NSCLC progression. Besides, genome-scale analysis to identify prognostic markers in patients with early stage pancreatic ductal adenocarcinoma after pancreaticoduodenectomy, found that *SLA2* is one of the prognostic markers [4]. The expression of *SLA2* in HNSCC was further verified, and the results showed that patients with higher *SLA2* expression had better prognosis. In addition, we found that *SLA2* gene expression was associated with tumor stage, clinical grade, and age. In addition, univariate and multivariate Cox regression analysis showed that *SLA2* expression was an independent prognostic factor for HNSCC. Therefore, *SLA2* has potential diagnostic value in HNSCC.

Next, we analyzed the *SLA2*-related pathway in HNSCC to understand its carcinogenic mechanism. Based on GO and KEGG analyses, we observed that the functional network of *SLA2* in HNSCC was related to the side of membrane, receptor complex, and other cellular component, where they were involved in biological processes, such as leukocyte cell–cell adhesion. These related genes also served as antigen binding, cytokine receptor activity, SH3 domain binding, and other molecular function cytokine receptor binding. Abplp is a yeast cortical actin binding protein that contains a SH3 domain similar to that found in signal transduction proteins that act at the membrane/cytoskeletal interface. Zero mutation of SLA1 and SLA2 leads to temperature sensitive growth defects. Sla1p contains three SH3 domains, which are essential for proper formation of cortical actin cytoskeleton [22]. KEGG pathway analysis indicated that these genes related to *SLA2* were mainly enriched in signal pathways, such as hematopoietic cell lineage, CAMs, and other pathway. *SLA2* is a recently discovered adaptor protein mainly expressed in hematopoietic cells. Studies shows that *SLA2* may have function as a negative regulator of GPVI-mediated signaling by interacting with c-Cbl, similar to what was reported in T cells [23]. All these results suggested that *SLA2* regulates immune effector process, and is involved in several related signaling pathways.

*SLA2* is a adapter protein, which negatively regulates TCR signaling. It inhibits T-cell antigen–receptor-induced activation of nuclear factor of activated T-cells. It may act by linking signaling proteins, such as ZAP70 with CBL, leading to a CBL dependent degradation of signaling proteins. The study also shows that *SLA2* is related to endosome membrane, which is involved in regulation of immune effector process [21]. In this study, we found that *SLA2* expression levels were positively correlated with the infiltration levels of numerous immune cells, suggesting that *SLA2* might promote the anti-tumor immune response in tumor tissues. Most malignancies increase levels of inhibitory ligands by impairing T cell function to avoid immune responses that lead to tumor growth. This is thought to be one of the potential mechanisms promoting tumor progression. Patients with higher levels of T-cell infiltration often have a better prognosis [24, 25]. In addition, *SLA2* expression levels were significantly correlated with the expression levels of many immune molecules, which might recruit immune cell infiltration, thereby exerting an anti-tumor effect. Altogether, these results suggest that *SLA2* may exert an anti-oncogenic function by triggering anti-tumor immune responses in HNSCC tissues.

Many reports support that immune cell infiltration in tumor tissues is related to tumor formation, growth and metastasis [26]. High concentration of immune promoting cells in tumor tissue can improve the prognosis of patients, while low concentration of immune promoting cells can lead to immune escape of cancer cells, resulting in poor prognosis [27]. When a tumor is strongly infiltrated by immune cells, the expression of SLA2 will increased, and strong infiltration by immune cells is associated with a more favourable prognosis. Our results are similar to the reported of Yang et al. [28].

To further evaluate the potential immune mechanisms of *SLA2* in HNSCC, we second analyzed *SLA2*-related immune infiltration levels. The results demonstrate that the *SLA2* expression level is strongly positive correlated with the infiltration level of many immune cells. After analyzing the correlation between *SLA2* expression and immune cell marker genes, the results revealed that the function of *SLA2* in regulating different immune infiltrating cells is different. *SLA2* had a positive relationship with most of the immune activation genes. In addition, the efficacy of immunotherapy not only requires sufficient immune cell infiltration in the tumor microenvironment, but also depends on the full expression of immune checkpoints [29]. The results demonstrated that *SLA2* positively co-expressed with these immune-checkpoints, indicating that *SLA2* may be a potential immunotherapy target. Moreover, cisplatin and methotrexate had better antitumor effect for HNSCC.

A large number of studies have confirmed that tumor immune cell infiltration can affect the efficacy and prognosis of chemotherapy, radiotherapy or immunotherapy in cancer patients [30, 31]. Taken together, SLA2 expression level is closely related to immune cell infiltration, which can lead to good prognosis of tumor patients. Most importantly, *SLA2* expression may affect the immune microenvironment and then indirectly affects the prognosis of HNSCC and serves as a predictor of ICB therapies and chemotherapeutics.However, the limitation of this study is that a large number of samples were needed to verify our results, and more clinical samples will be collected to enrich the data in the future. Moreover, the underlying immune mechanisms should be explored and *SLA2* as biomarkers to predict the immune response rate in real-world HNSCC patients should be conducted.

In conclusion, our work demonstrated that *w*hen a tumor is strongly infiltrated by immune cells, the expression of SLA2 will increased, and strong infiltration by immune cells is associated with a more favourable prognosis. *SLA2* may be a potential target for immunotherapy.

## Data Availability

The data in this study are available from the corresponding author upon request.

## Abbreviations

aDC: Activated dendritic cell
BP: Biological process
CAMs: Cell adhesion molecules
CBL: Casitas B-lineage lymphoma proto-oncogene
CC: Cellular component
CD274: Cluster of differentiation 274
CD4 + T cells: Cluster of differentiation 4-positive T cells
CD8 T cells: Cluster of differentiation 8 T cells
CD8 + T cells: Cluster of differentiation 8-positive T cells
CD28: Cluster of differentiation 28 T cells
CD48: Cluster of differentiation 48 T cells
CXCR4: C–X–C motif chemokine receptor 4
CTLA4: Cytotoxic T lymphocyte-associated antigen-4
DC: Dendritic cell
DSS: Disease-specific survival
GDSC: The genomics of drug sensitivity in cancer
GEPIA: Gene expression profile interaction analysis
GO: Gene ontology
GSEA: Gene set enrichment analysis
GSVA: Gene set variation analysis
GTeX: The genotype-tissue expression
HAVCR2: Hepatitis A virus cellular receptor 2
HNSCC: Head and neck squamous cell carcinoma
HR: Hazard ratio
ICB: Immune checkpoint blockade
IC50: The samples’ half-maximal inhibitory concentration
iDC: Interdigitating dendritic cell
IL6: Interleukin-6
KEGG: Kyoto encyclopedia of genes and genomes
KIRC: Kidney renal clear cell carcinoma
KIRP: Kidney renal papillary cell carcinoma
KM: Kaplan–Meier
LAG3: Lymphocyte-activation gene 3
LCK: Lymphocyte-specific protein tyrosine kinase
MF: Molecular function
MHC genes: Major histocompatibility complex gene
mRNA: Messenger ribonucleic acid
NK CD56dim cells: Natural killer CD56dim cells
RNA-seq: Ribonucleic acid sequencing
SCC: Squamous cell carcinoma
SH3: Src homology domain 3
SIGLEC15: Sialic acid binding Ig-like lectin 15
SLA2: Src-like adaptor 2 gene
SsGSEA: Single-sample gene set enrichment analysis
STAD: Stomach adenocarcinoma
TCGA: The cancer genome atlas
TCR: T-cell receptor
Tcm: Central memory T cells
Tem: Effective memory T cell
TFH: Follicular helper T cell
Th1 cells: T helper 1 cells
Th2 cells: T helper 2 cells
TIGIT: T cell immunoreceptor with Ig and ITIM domains
TIICs: Tumor-infiltrating immune cells
TIMER: Tumor Immune Estimation Resource
TME: Tumor microenvironment
TPM: Transcripts per million reads
TReg: Regulatory T cell
OS: Overall survival
pDC: Plasmacytoid dendritic cells
PDCD1: Programmed cell death 1
PDCD1LG2: Programmed cell death 1 ligand 2
WHO: World Health Organization
ZAP70: Zeta chain of T cell receptor-associated protein kinase 70
95% CI: 95% Confidence intervals

## References

[1] Sun X, Zhang L, Liu S (2021). The immune infiltration in hnscc and its clinical value: a comprehensive study based on the tcga and geo databases. Comput Math Methods Med.

[2] Meng Y, Huang T, Chen X, Lu Y (2021). A comprehensive analysis of the expression and regulation network of lymphocyte-specific protein tyrosine kinase in breast cancer. Transl Cancer Res..

[3] Li J, Bi L, Shi Z, Sun Y, Lin Y, Shao H, Zhu Z (2016). Rna-seq analysis of non-small cell lung cancer in female never-smokers reveals candidate cancer-associated long non-coding rnas. Pathol Res Pract.

[4] Liao X, Huang K, Huang R, Liu X, Han C, Yu L, Yu T, Yang C, Wang X, Peng T (2017). Genome-scale analysis to identify prognostic markers in patients with early-stage pancreatic ductal adenocarcinoma after pancreaticoduodenectomy. Onco Targets Ther.

[5] Cohen EEW, Bell RB, Bifulco CB, Burtness B, Gillison ML, Harrington KJ, Le QT, Lee NY, Leidner R, Lewis RL, Licitra L, Mehanna H, Mell LK, Raben A, Sikora AG, Uppaluri R, Whitworth F, Zandberg DP, Ferris RL (2019). The society for immunotherapy of cancer consensus statement on immunotherapy for the treatment of squamous cell carcinoma of the head and neck (hnscc). J Immunother Cancer.

[6] Wang G, Zhang M, Cheng M, Wang X, Li K, Chen J, Chen Z, Chen S, Chen J, Xiong G, Xu X, Wang C, Chen D (2021). Tumor microenvironment in head and neck squamous cell carcinoma: functions and regulatory mechanisms. Cancer Lett.

[7] Jung AR, Jung CH, Noh JK, Lee YC, Eun YG (2020). Epithelial-mesenchymal transition gene signature is associated with prognosis and tumor microenvironment in head and neck squamous cell carcinoma. Sci Rep.

[8] Vivian J, Rao AA, Nothaft FA, Ketchum C, Armstrong J, Novak A, Pfeil J, Narkizian J, Deran AD, Musselman-Brown A, Schmidt H, Amstutz P, Craft B, Goldman M, Rosenbloom K, Cline M, O'Connor B, Hanna M, Birger C, Kent WJ, Patterson DA, Joseph AD, Zhu J, Zaranek S, Getz G, Haussler D, Paten B (2017). Toil enables reproducible, open source, big biomedical data analyses. Nat Biotechnol.

[9] Smyth GK, Michaud J, Scott HS (2005). Use of within-array replicate spots for assessing differential expression in microarray experiments. Bioinformatics.

[10] Tang Z, Li C, Kang B, Gao G, Li C, Zhang Z (2017). Gepia: a web server for cancer and normal gene expression profiling and interactive analyses. Nucleic Acids Res.

[11] Hänzelmann S, Castelo R, Guinney J (2013). Gsva: gene set variation analysis for microarray and rna-seq data. BMC Bioinformatics.

[12] Bindea G, Mlecnik B, Tosolini M, Kirilovsky A, Waldner M, Obenauf AC, Angell H, Fredriksen T, Lafontaine L, Berger A, Bruneval P, Fridman WH, Becker C, Pagès F, Speicher MR, Trajanoski Z, Galon J (2013). Spatiotemporal dynamics of intratumoral immune cells reveal the immune landscape in human cancer. Immunity.

[13] Li T, Fan J, Wang B, Traugh N, Chen Q, Liu JS, Li B, Liu XS (2017). Timer: a web server for comprehensive analysis of tumor-infiltrating immune cells. Cancer Res.

[14] Jiang P, Gu S, Pan D, Fu J, Sahu A, Hu X, Li Z, Traugh N, Bu X, Li B, Liu J, Freeman GJ, Brown MA, Wucherpfennig KW, Liu XS (2018). Signatures of t cell dysfunction and exclusion predict cancer immunotherapy response. Nat Med.

[15] Lu X, Jiang L, Zhang L, Zhu Y, Hu W, Wang J, Ruan X, Xu Z, Meng X, Gao J, Su X, Yan F (2019). Immune signature-based subtypes of cervical squamous cell carcinoma tightly associated with human papillomavirus type 16 expression, molecular features, and clinical outcome. Neoplasia.

[16] Geeleher P, Cox NJ, Huang RS (2014). Clinical drug response can be predicted using baseline gene expression levels and in vitro drug sensitivity in cell lines. Genome Biol.

[17] Jiang Q, Sun J, Chen H, Ding C, Tang Z, Ruan Y, Liu F, Sun Y (2021). Establishment of an immune cell infiltration score to help predict the prognosis and chemotherapy responsiveness of gastric cancer patients. Front Oncol.

[18] Vasaikar SV, Straub P, Wang J, Zhang B (2018). Linkedomics: analyzing multi-omics data within and across 32 cancer types. Nucleic Acids Res.

[19] Zhou LQ, Hu Y, Xiao HJ (2021). The prognostic significance of survivin expression in patients with hnscc: a systematic review and meta-analysis. BMC Cancer.

[20] Oliva M, Spreafico A, Taberna M, Alemany L, Coburn B, Mesia R, Siu LL (2019). Immune biomarkers of response to immune-checkpoint inhibitors in head and neck squamous cell carcinoma. Ann Oncol.

[21] Holland SJ, Liao XC, Mendenhall MK, Zhou X, Pardo J, Chu P, Spencer C, Fu A, Sheng N, Yu P, Pali E, Nagin A, Shen M, Yu S, Chan E, Wu X, Li C, Woisetschlager M, Aversa G, Kolbinger F, Bennett MK, Molineaux S, Luo Y, Payan DG, Mancebo HS, Wu J (2001). Functional cloning of src-like adapter protein-2 (slap-2), a novel inhibitor of antigen receptor signaling. J Exp Med.

[22] Holtzman DA, Yang S, Drubin DG (1993). Synthetic-lethal interactions identify two novel genes, sla1 and sla2, that control membrane cytoskeleton assembly in saccharomyces cerevisiae. J Cell Biol.

[23] Sugihara S, Katsutani S, Deckmyn H, Fujimura K, Kimura A (2010). Roles of src-like adaptor protein 2 (slap-2) in gpvi-mediated platelet activation slap-2 and gpvi signaling. Thromb Res.

[24] Oh DY, Kwek SS, Raju SS, Li T, McCarthy E, Chow E, Aran D, Ilano A, Pai CS, Rancan C, Allaire K, Burra A, Sun Y, Spitzer MH, Mangul S, Porten S, Meng MV, Friedlander TW, Ye CJ, Fong L (2020). Intratumoral cd4(+) t cells mediate anti-tumor cytotoxicity in human bladder cancer. Cell.

[25] van der Leun AM, Thommen DS, Schumacher TN (2020). Cd8(+) t cell states in human cancer: Insights from single-cell analysis. Nat Rev Cancer.

[26] Ringgaard L, Melander F, Eliasen R, Henriksen JR, Jølck RI, Engel TB, Bak M, Fliedner FP, Kristensen K, Elema DR, Kjaer A, Hansen AE, Andresen TL (2020). Tumor repolarization by an advanced liposomal drug delivery system provides a potent new approach for chemo-immunotherapy. Sci Adv.

[27] Chen H, Xie J, Jin P (2020). Assessment of hazard immune-related genes and tumor immune infiltrations in renal cell carcinoma. Am J Transl Res.

[28] Yang Z, Tian H, Bie F, Xu J, Zhou Z, Yang J, Li R, Peng Y, Bai G, Tian Y, Chen Y, Liu L, Fan T, Xiao C, Zheng Y, Zheng B, Wang J, Li C, Gao S, He J (2021). ERAP2 is associated with immune infiltration and predicts favorable prognosis in SqCLC. Front Immunol.

[29] Chae YK, Arya A, Iams W, Cruz MR, Chandra S, Choi J, Giles F (2018). Current landscape and future of dual anti-ctla4 and pd-1/pd-l1 blockade immunotherapy in cancer; lessons learned from clinical trials with melanoma and non-small cell lung cancer (nsclc). J Immunother Cancer.

[30] Zhang H, Liu H, Shen Z, Lin C, Wang X, Qin J, Qin X, Xu J, Sun Y (2018). Tumor-infiltrating neutrophils is prognostic and predictive for postoperative adjuvant chemotherapy benefit in patients with gastric cancer. Ann Surg.

[31] Lyu L, Yao J, Wang M, Zheng Y, Xu P, Wang S, Zhang D, Deng Y, Wu Y, Yang S, Lyu J, Guan F, Dai Z (2020). Overexpressed pseudogene hla-dpb2 promotes tumor immune infiltrates by regulating hla-dpb1 and indicates a better prognosis in breast cancer. Front Oncol.

